# Correlation of textile ‘resistance to damage’ scores with actual physical survival of long-lasting insecticidal nets in the field

**DOI:** 10.1186/s12936-020-03570-5

**Published:** 2021-01-07

**Authors:** Albert Kilian, Emmanuel Obi, Paul Mansiangi, Ana Paula Abílio, Khamis Ameir Haji, Estelle Guillemois, Vera Chetty, Amy Wheldrake, Sean Blaufuss, Bolanje Olapeju, Stella Babalola, Stephen J. Russell, Hannah Koenker

**Affiliations:** 1 PMI VectorWorks Project, Tropical Health LLP, Montagut, Spain; 2 PMI VectorWorks Project, Tropical Health LLP, Abuja, Nigeria; 3 Ecole de Santé Publique, Université de Kinshasa, Kinshasa, Democratic Republic of the Congo; 4 Instituto Nacional de Saúde, Maputo, Mozambique; 5 Zanzibar Malaria Elimination Programme, Stone Town, Zanzibar, Tanzania; 6 Nonwovens Innovation and Research Institute Ltd. (NIRI), Leeds, UK; 7 PMI VectorWorks Project, JHU Center for Communication Programs, Baltimore, MD USA

**Keywords:** LLIN physical durability, Textile resistance to damage

## Abstract

**Background:**

Attempts have been made to link procurement of long-lasting insecticidal nets (LLIN) not only to the price but also the expected performance of the product. However, to date it has not been possible to identify a specific textile characteristic that predicts physical durability in the field. The recently developed resistance to damage (RD) score could provide such a metric. This study uses pooled data from durability monitoring to explore the usefulness of the RD methodology.

**Methods:**

Data from standardized, 3-year, prospective LLIN durability monitoring for six LLIN brands in 10 locations and four countries involving 4672 campaign LLIN were linked to the RD scores of the respective LLIN brands. The RD score is a single quantitative metric based on a suite of standardized textile tests which in turn build on the mechanisms of damage to a mosquito net. Potential RD values range from 0 to 100 where 100 represents optimal resistance to expected day-to-day stress during reasonable net use. Survival analysis was set so that risk of failure only started when nets were first hung. Cox regression was applied to explore RD effects on physical survival adjusting for known net use environment variables.

**Results:**

In a bivariate analysis RD scores showed a linear relationship with physical integrity suggesting that the proportion of LLIN with moderate damage decreased by 3%-points for each 10-point increase of the RD score (p = 0.02, R^2^ = 0.65). Full adjustment for net care and handling behaviours as well as other relevant determinants and the country of study showed that increasing RD score by 10 points resulted in a 36% reduction of risk of failure to survive in serviceable condition (p < 0.0001). LLINs with RD scores above 50 had an additional useful life of 7 months.

**Conclusions:**

This study provides proof of principle that the RD metric can predict physical durability of LLIN products in the field and could be used to assess new products and guide manufacturers in creating improved products. However, additional validation from other field data, particularly for next generation LLIN, will be required before the RD score can be included in procurement decisions for LLINs.

## Background

The development of the long-lasting insecticidal net (LLIN) technology was crucial in making insecticide-treated mosquito nets (ITN) the primary tool for malaria prevention in Africa it is today. Initially, only two brands were available, the polyethylene-based Olyset™ Net which obtained an interim recommendation for public health use from the World Health Organization (WHO) Pesticide Evaluation Scheme (WHOPES) in 2001 [1] and the polyester-based PermaNet^®^ 2.0 which obtained interim WHOPES recommendation in 2003 [2]. However, as the implementation of LLIN mass distribution campaigns picked up after this approach had been shown to provide high and equitable coverage [3], more LLIN brands came on the market. By 2007 two polyethylene-based LLIN products, DuraNet© and Netprotect^®^ [4] and one polyester-based brand, Interceptor^®^ [5] had received interim WHOPES recommendations and by end of 2015 there were 13 pyrethroid LLIN brands with at least interim WHOPES recommendation (excluding LLINs with the synergist piperonyl butoxide (PBO) to counter increasing insecticide resistance against pyrethroids), six polyester-based and seven polyethylene-based [6–8]. Some of these LLIN products never made it to the market such as the polypropylene-based Lifenet^®^, which combined the softer textile structure of polyester, often preferred by consumers, with the strength of a monofilament yarn similar to polyethylene. But the product could not compete with other LLIN brands due to the higher price of the material. Others are products that share the same specifications and received WHOPES recommendation by extension of recommendations previously given to the “original” product. This applies to MAGNet^®^ and Royal Sentry^®^ [7] which are based on the DuraNet© specification, Yorkool^®^ [6] and Yahe^®^ [8] based on PermaNet^®^ 2.0, and SafeNet^®^ [8] based on the Interceptor^®^ LLIN. Since 2017, evaluation of vector control products is centralized with WHO’s Prequalification Team and as of August 2020 the list of prequalified LLIN products includes 13 conventional pyrethroid LLINs and eight “next generation” LLIN products with either the synergist PBO added or a second active ingredient [9].

With increasing demand for public health use of LLIN and increasing competition among manufacturers, prices not only significantly decreased over time (in part due to reduced prices of oil-based raw materials), but also varied significantly between products [10]. This then raised the question whether any specific LLIN brand might have a better “value for money” or “cost per year of useful life” than another brand, i.e. considering not only the absolute price per unit, but also the insecticidal and physical durability of the product in the field. While this notion was widely accepted in general and included in the procurement guidelines for pesticide products by the WHO [11], its implementation was hindered by the fact that the initial methodology for field testing LLINs focused on the insecticidal effectiveness and aspects of physical decay were poorly understood [12]. This changed in 2013 with the approval of a revised methodology for monitoring physical LLIN durability in the field by the Malaria Policy Advisory Committee (MPAC) that combined attrition (loss) of nets due to discarding or destruction with a robust and standardized measure of integrity of surviving nets [13].

Available data on the physical survival of LLIN in the field show a wide variation in “useful life” between locations or in different use environments ranging from less than 2 years [14, 15] to four or more [16–19]. Some limited data exists comparing performance of two or more LLIN brands in the same or similar use environments and the results are mixed. Some found differences [15, 20–23] while others did not [24, 25]. A recent multi-country and multi-brand secondary analysis of data from the VectorWorks project funded by the U.S. President’s Malaria Initiative suggests that a simple categorization by material (polyethylene vs. polyester) and weight of yarn (denier) is not sufficient to describe performance differences between LLIN brands and a more nuanced metric is needed which better reflects the mechanisms of mosquito net damage under “normal” or reasonable day-to-day use [26]. Such a composite textile performance metric has now been proposed as the resistance to damage (RD) score [27]. This study uses the previously mentioned VectorWorks-generated data to explore whether the RD score obtained from pre-distribution textile testing can be used as a predictor for LLIN field performance adjusting for other elements of net use environment.

## Methods

### Study sites

Data from 10 sites of durability monitoring activities in four countries involving six LLIN brands were included in the analysis and details of locations and LLIN brands studied are shown in Table 1. There were two distinct country scenarios. In Mozambique and Nigeria, the same or similar LLIN brands were tested in what was expected to be different net use environments, while in the Democratic Republic of Congo (DRC) and Zanzibar different LLIN brands were monitored in similar locations. The selection of the scenario was based on the information needs of the malaria programme.

**Table 1:** Countries, locations, LLIN brands and sample size

Country	Province (State)	District (Local Government Area, Health Zone,)	LLIN brand	LLIN in cohort study
Mozambique	Inhambane	Jangamo	Royal Sentry^®^	726
Mozambique	Nampula	Angoche	Royal Sentry^®^	661
Mozambique	Tete	Changara	MAGNet^®^	601
Nigeria	Ebonyi	Ishielu	DawaPlus 2.0^®^	367
Nigeria	Oyo	Akinyele	DawaPlus 2.0^®^	372
Nigeria	Zamfara	Bakura	DawaPlus 2.0^®^	357
Democratic Republic Congo	Mongala	Binga	DawaPlus 2.0^®^	377
Democratic Republic Congo	Ubangi Sud	Ndege	DuraNet©	377
Zanzibar (Tanzania)	Pemba	Wete	Olyset™ Net	452
Zanzibar (Tanzania)	Unguja	North B	PermaNet 2.0^®^	382

### Primary data collections

The study design was the same in all countries and followed a standardized protocol recommended by the PMI [28] and in line with WHO recommendations [13]. Details of the methodology and used tools have been presented previously [19]. In short, a representative sample of LLINs distributed through a mass distribution campaign organized by the respective malaria programmes were recruited into a prospective cohort study 1 to 6 months after distribution. Sample size target was 345 cohort nets per site (district or equivalent) sampled from 15 clusters per site and 10 households per cluster in Nigeria, Zanzibar and DRC. In Mozambique, the sample size was higher with 782 cohort nets targeted per site from 17 clusters. These differences were due to varying assumptions for precision of estimates. Clusters were selected with probability proportionate to size and households were selected by simple random sampling from lists prepared at the day of the survey. Follow-up surveys were conducted approximately 12, 24 and 36 months after distribution. At each time point presence or loss of the nets as well as reasons for losses were recorded (attrition) and an assessment of the physical integrity of the remaining cohort nets was carried out. Data collections took place between November 2015 and April 2019. Follow-up in Oyo State, Nigeria only was for 24 months due to a delay in the LLIN mass campaign and the end of the VectorWorks project. All other sites completed the 36 months follow-up survey. Data was collected electronically using tablets and the Open Data Kit (ODK) software. After data cleaning and consistency checks data was transferred to the Stata statistical package (Stata version 14.2, College Station, Texas, USA) for processing and analysis.

Physical integrity was measured by the proportionate Hole Index (pHI) as recommended by the WHO [29] and then categorized based on the pHI value as still serviceable (pHI ≤ 642) or torn (pHI > 642) [30]. Primary outcome of the physical durability assessment was the survival in serviceable condition which incorporates attrition due to discarding of nets (destroyed, thrown away or used for other purposes) and surviving nets no longer serviceable. Nets that were given to others to use or for which outcome was unknown were excluded from the uni- and bivariate analysis and censored in the survival analysis [29].

In addition, information on socio-demographic characteristics, ownership of other mosquito nets, net use environment, net handling, and net care and repair behaviour was collected through household-level questionnaires. Specifically, a household net care attitude variable was developed based on Likert scale comprising six questions with a four-value response, omitting the neutral option. Based on this variable households were categorized as never, sometimes or always showing very positive net care attitude (score ≥ 1.0 from a range − 2.0 to 2.0) across the up to four surveys each household participated in [19]. Similarly, a variable of household exposure to social and behaviour change (SBC) messages regarding LLIN was created with categories of being exposed “never”, “at least once” or “twice or more” during the course of the study.

### Resistance to damage scores

The RD score is a single quantitative metric based on a suite of modified, standardized textile tests which in turn build on the study of the mechanisms that lead to damage of mosquito nets. The methodology has been presented in detail previously [27]. In short, the RD score can take any value between 0 and 100 and considers both human factors and laboratory testing data. Quantitative reference forces applied to LLINs by users during normal use were determined so that aspirational performance levels could be established for each of four textile tests. The RD scores for the LLIN products used in this study were based on an algorithm that uses a proximity to these aspirational values. In this method, the actual values of laboratory testing data for snag strength, bursting strength, abrasion and hole enlargement were compared with aspirational values for each parameter to determine numerical differences in performance. The mean value for each parameter was then divided by four so that each contributed equally to the overall RD value, expressed as a percentage. RD score results were obtained from a sample of ex-factory LLIN tested at the Nonwovens Innovation & Research Institute (NIRI), Leeds, UK independent of the distribution of LLIN for the campaigns used in the field data. Details of the testing results have been described elsewhere [27].

### Secondary data analysis

For this secondary analysis four types of data sets were used from each country, the household and cohort LLIN master lists and the household and cohort net result file including all observations across all four surveys per site. These data sets were then merged and unique identifiers created for each cohort net and household within each site. To each of the net data sets the RD value for the LLIN of the specific site was added and a group variable for above and below a RD value of 50 (midpoint) was created.

Based on the findings of the separate country data analyses on the relationship between SBC message exposure and net care attitude a new variable was created for the secondary analysis that combined the two variables into four groups as follows: (i) never positive net care attitude and never SBC exposed; (ii) never positive net care attitude and one or more SBC exposures; (iii) at least one positive care attitude combined with any number of SBC exposures; (iv) at least twice positive net care attitude and at least twice exposed to SBC messages.

### Statistical analysis

Data was set up for survival analysis as a duration format data set where each time interval for a net was a separate observation. Survival analysis was done using a per-protocol approach, i.e. risk of failure was considered to start only on the first observation where the net was found hanging, i.e. excluding any net that was never hung as well as the time period to first hanging. Failure was defined as a net being reported lost to wear and tear or torn based on physical assessment (pHI). The time of failure was directly calculated from the reported time of loss by the respondent or taken as the mid-point between the last two surveys if time of loss was unknown.

For continuous variables, arithmetic means were used to describe the central tendency and the t-test for comparison of groups for normally distributed data. Otherwise, median and Kruskal–Wallis test were used. Proportions were compared by contingency tables and the Chi squared test used to test for differences in proportions. For calculation of confidence intervals around estimates, the intra- and between-cluster correlation has been taken into account using the *svy* command in Stata.

Determinants of survival in serviceable condition after the net was first hung were explored using Cox proportionate hazard models. Factors were tested first in individual models which were then used to construct the final multivariate models. Final model fit was tested using a linktest and Schoenfeld residuals and log–log plots were used to check the proportionate hazard assumption.

## Results

A total of 4672 campaign nets from 1976 households were recruited into the cohorts at each site (details see Table 1) and for 75% of these definite outcomes of physical durability could be determined in the course of the studies. In addition, a total of 7545 observations on physical integrity of surviving nets were made, 3489 at the 12-months survey, 2499 at the 24-, and 1557 at the 36-months survey. The LLIN brands with the most observations were DawaPlus^®^ 2.0 with 2404 (32%) and Royal Sentry^®^ with 2080 (28%) as both were distributed in multiple sites.

Four brands represented polyethylene (PET) and two polyester (PE) based LLIN and general textile characteristics were identical within each of these two groups (Table 2). The RD scores as determined in the lab were found to be a narrow range, from 29 to 63. Three LLIN brand scored RD < 50 and three RD > 50. The three LLIN brands with RD scores above 50 all belong to the PE group of LLIN but the fourth brand in this group actually had the lowest RD value of only 29. Of the LLINs recruited into the cohorts 50% were in the > 50 RD score group and of the observations on integrity of surviving nets beyond baseline 47% fell into this group.

**Table 2:** Textile characteristics and RD scores of LLIN brands

LLIN brand	Material	Yarn mass per length (denier)	Yarn type	RD score value
Brand A	PET	100	Multi-filament	39
Brand B	PET	100	Multi-filament	41
Brand C	PE	150	Mono-filament	55
Brand D	PE	150	Mono-filament	63
Brand E	PE	150	Mono-filament	52
Brand F	PE	150	Mono-filament	29

PE: polyester; PET: polyethylene

**Fig. 1:**
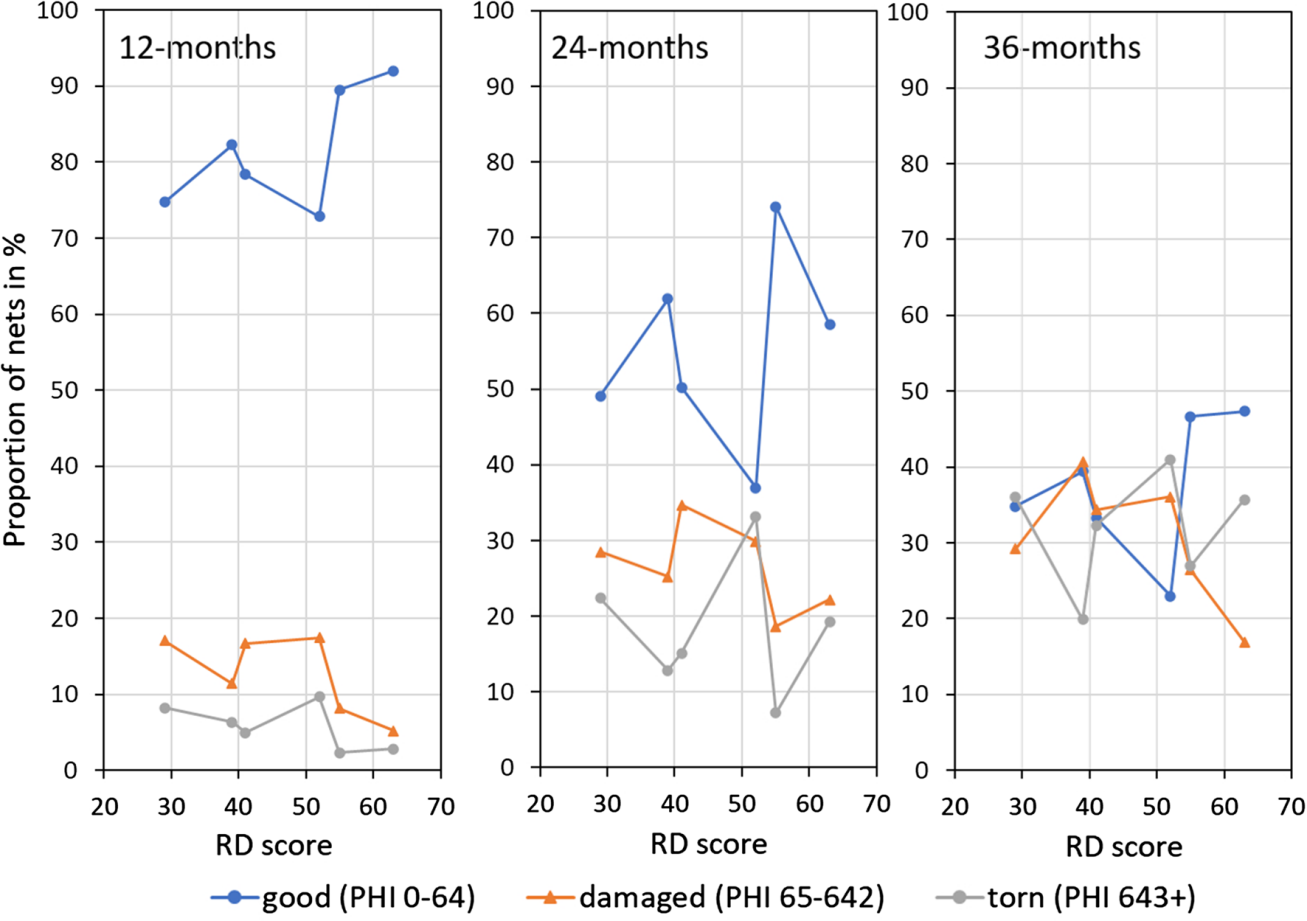
Physical condition of surviving LLIN by RD score and time of follow-up

The physical condition of surviving LLIN, grouped as good, damaged or torn, is shown as a function of the RD score in Fig. 1 for each of the three follow-up surveys. At 12 months there was a reasonable linear relationship with the RD scores in the anticipated direction for all three groups, i.e. increasing proportion of good nets and decreasing for damaged and torn nets as RD scores increase. The one outlier was an LLIN brand for which only one data point existed and where the country analysis had shown generally very poor performance of both LLIN brands tested there [15]. Without that data point, all three pHI groups showed a significant gradient in a bivariate linear regression (p = 0.03). With increasing time, the crude relationship between physical condition and RD scores became less evident and in the pooled linear regression adjusting for time of survey only the damaged category showed a significant relationship with the RD scores (coefficient − 0.31, 95% CI − 0.57 to − 0.05, R^2^ 0.65, p = 0.02).

**Fig. 2:**
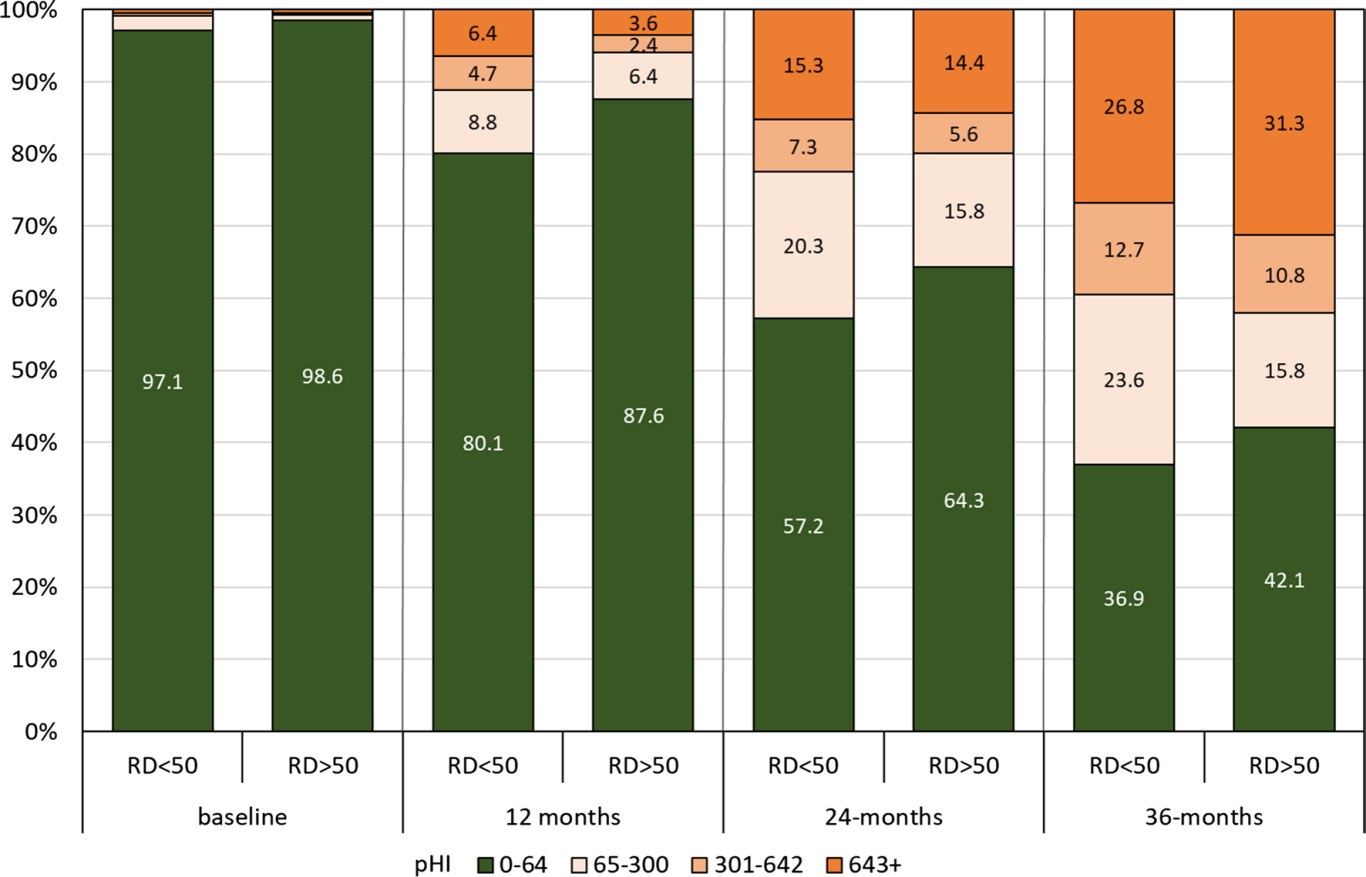
Severity of damage in surviving LLIN over time comparing LLIN below and above RD score of 50

Further splitting the damaged group into two (pHI 65–300 and 301–642) and using the above and below RD 50 grouping for the LLIN brands for comparison shows that LLINs with an RD score above 50 had less damage at 12-months (p = 0.0001), 24-months (p = 0.03) and 36-months (p = 0.02) follow-up (Fig. 2).

The country by country analysis had revealed a number of variables describing net use environment and net care as significant determinants of survival of LLIN in serviceable condition [15, 19, 23, 31] and a recent pooled analysis has confirmed that some of these are strong predictors across countries [26]. Using these previously established determinants in a Cox regression of LLIN survival (Table 3) shows a significant protective effect of the RD metric suggesting that every increase of the RD score by 10 points reduced the hazard of failure by 36% (adjusted hazard ratio (aHR) 0.64). When the below/above RD 50 was used, the model suggests reduction of the risk of failure by half for the > 50 RD score group (aHR 0.46, 95% CI 0.35–0.60, p < 0.0001).

**Table 3:** Determinants of physical survival of LLIN in a per-protocol Cox regression model

Variable	Adjusted Hazard Ratio (aHR)	95% CI	p-value
N = 5126 obs/2900 nets			
High net care attitude score and SBC exposure			
Attitude never–SBC never	1.00		
Attitude never–SBC at least once	0.66	0.52–0.83	< 0.0001
Attitude at least once–SBC never or at least once	0.56	0.45–0.70	< 0.0001
Attitude at least twice–SBC at least twice	0.35	0.26–0.46	< 0.0001
RD value/10 (impact of increase by 10 points of RD)	0.64	0.55–0.74	< 0.0001
Never folding net up during day when hanging	1.41	1.18–1.69	< 0.0001
Never cooking inside the sleeping room	0.79	0.69–0.91	0.001
Dominant net users			
Child only	1.00		
Child with adult	0.86	0.70–1.05	0.14
Adult only	0.69	0.57–0.83	< 0.0001
Wealth tertile			
Lowest	1.00		
Middle	0.94	0.80–1.10	0.44
Highest	0.84	0.71–0.99	0.04
Gender of head of household			
Male	1.00		
Female	0.84	0.67–1.04	0.12
Country			
Mozambique	1.00		
Nigeria	0.30	0.21–0.43	< 0.0001
DRC	1.29	1.01–1.65	0.05
Zanzibar (Tz)	0.50	0.34–0.73	< 0.0001

Interestingly, if only the LLIN material, i.e. polyester (reference) vs. polyethylene is used, there was no effect with aHR 0.94 (95% CI 0.77–1.15, p = 0.58). The only difference between the RD 50 and PE/PET groups was one PE LLIN brand with a low RD score of 29. Removing this particular net from the PE group then only left PE LLINs with RD scores above 50 and resulted in a similar hazard ratio as the RD group (aHR 0.45, p < 0.0001).

**Fig. 3:**
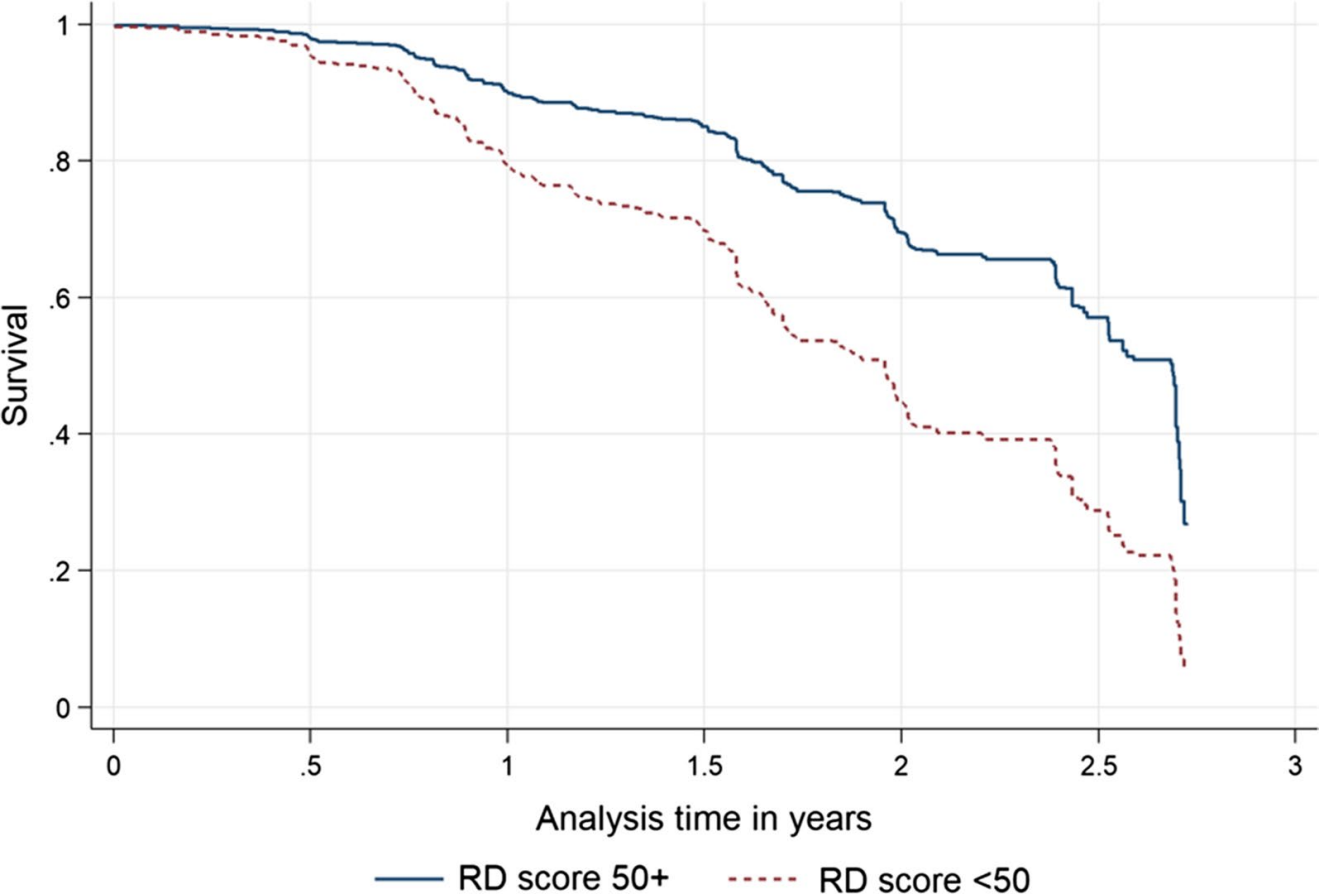
Adjusted survival curves of LLIN below and above RD score of 50

Finally, the survival curve from the Cox regression model adjusting for net use environment and net handling and care as well as country was plotted for the RD 50 group and the result is shown in Fig. 3. As early as 6 months after LLINs have first been hung, a better survival for LLIN products with RD score larger than 50 is visible. The time point at which 50% survival is reached is approximately 7 months later for the products with higher RD score.

## Discussion

The purpose of this study was to explore the usefulness of the recently suggested resistance to damage (RD) score as a tool to predict physical performance of LLIN products in the field [27] and, thereby, allow selection of products based on value for money as proposed by the WHO procurement guideline [11] rather than price alone. Using the pooled data set of 4672 campaign nets recruited into prospective cohort studies at 10 sites in four African countries allowed to effectively test the hypothesis that products with a higher RD score do, indeed, offer a longer survival in serviceable condition in the field, because it provided standardized and detailed data on net use environment and net care required to adjust for variations in local use conditions which otherwise could have obscured effects of the RD [32]. The RD scores of the six LLIN brands represented in the study varied from 29 to 63 reflecting the previous finding that current LLIN products not only offer limited variation in RD scores, but also are far from the aspirational level of 100 that represents optimal resistance to hole formation and propagation under reasonable day-to-day use [27].

The bivariate analysis of physical integrity (grouped pHI) against a continuous RD variable showed a linear correlation that was strongest at the 12-months follow-up, i.e. early on in the process of hole development and most pronounced for moderate damage (pHI 65–642) rather than for torn nets (pHI 643+). This is plausible because the “torn” group of integrity assessment is the exit category when nets are discarded in one way or another because of their damage and this happens at different levels of damage in different environments [33, 34], thus diluting the RD effect. There was good statistical evidence that, adjusting for time of survey, the proportion of LLIN with moderate damage decreased by 3 percentage-points for every 10-point increase of the RD score (p = 0.02), but there also was considerable variation or outliers due to significant differences in survival estimates for LLIN between countries, especially Nigeria and DRC [15, 19].

In order to adjust for variation in net use environment and net care behaviours a multivariate survival analysis was done using a per-protocol approach where risk of failure only starts with first observed hanging of a net in order to exclude effects of late or non-use of campaign nets. The final Cox regression model not only showed that a high level of net care attitude in combination with multiple exposures to SBC messages was the strongest determinant of LLIN physical durability (discussed in detail in [26]), but also that after adjustment for net use environment and country, there was a strong effect of the RD score on survival of the LLIN suggesting that every 10-point increase of the RD score reduces the hazard of failure to survive by 36% (p < 0.0001). When LLIN products were grouped around the median point of the RD scale, i.e. below and above RD of 50, the adjusted survival curves suggest that LLIN brands with an RD score above 50 gain approximately 7 months of useful life. Considering that this group only represented RD scores in the range of 52–63, this suggests that an innovative LLIN product with an RD score of 80 or more could have an advantage of more than 1 year in useful life and even at a higher cost would still have a lower equivalent annual cost [22].

Previous work has suggested that based on their textile characteristics polyethylene-based LLIN can be expected to be stronger than polyester-based products [35]. However, this was not the case in this study. When polyethylene LLINs were compared to polyester LLINs in the adjusted Cox regression, no evidence of an effect on survival was seen. There were four polyethylene LLIN in the study, three of which had an RD score above 50 and one significantly below 50 with 29, the lowest RD value in study. Once the brand with the lowest RD score was removed from the data set the effect of polyethylene vs. polyester in the Cox regression was similar to the RD group comparison. This suggests that LLIN have characteristics that result in faster and more severe hole development in the field which are not captured by the material category, but are reflected by the RD score which includes snag and bursting strength as well as resistance to abrasion and hole enlargement [27]. The potential causes of such an effect in terms of how the net is designed, and how it could be improved is a relevant question but beyond the scope of this study.

This study is limited as the selection of LLIN brands included was opportunistic, depending on which brands happened to be distributed in the countries where the project supported the LLIN durability activities. Furthermore, only two of the LLIN brands were studied in more than one location. This could have influenced the comparability in bivariate analysis, i.e. without adjustment for variables of net use environment and location. Ideally a multi-brand, multi-location comparison of LLIN physical durability would be designed as random distribution of the same LLIN products in each of multiple and diverse locations, but such a study does not exist to date. Even the recently published multi-brand, multi-country analysis by Briët et al. includes some countries with only a single brand and the list of LLIN products differed between countries [32]. On the other hand, the data in this study included the most comprehensive set of standardized covariates to date on net use environment and net care behaviours, allowing for adjustments that in essence created a standardized field use environment against which the capacity of the RD metric could be tested. Furthermore, the survival analysis, on which the major conclusion is based, used a per-protocol approach that defines the first observed hanging of the net as the start for the risk of failure, thereby excluding any impact that differences in initial utilization of the campaign nets could have had.

Another limitation is the fact that RD scores were determined from ex-factory LLIN samples provided by manufacturers and it cannot be excluded that the RD scores of the products actually delivered to national malaria control programmes may have deviated from these values due to variations in production quality.

## Conclusions

The results of this study provide proof of principle that the resistance to damage metric can predict physical durability of LLIN products in the field and could be used as a guidance to assess new products and guide manufacturers in creating improved products. However, additional validation from other field data, particularly for next generation LLIN, will be required before the RD score can be expected to be included into procurement procedure for LLIN. Such studies should involve the same group of LLIN brands distributed in multiple locations with a rigorous assessment of and adjustment for net use environment and behaviours.

## Data Availability

The datasets used and/or analysed during the current study are available from the corresponding author on reasonable request.
